# Autophagy orchestrates the crosstalk between cells and organs

**DOI:** 10.15252/embr.202357289

**Published:** 2023-07-19

**Authors:** Klara Piletic, Ghada Alsaleh, Anna Katharina Simon

**Affiliations:** ^1^ Kennedy Institute of Rheumatology University of Oxford Oxford UK; ^2^ Botnar Institute for Musculoskeletal Sciences, NDORMS University of Oxford Oxford UK; ^3^ Max Delbrück Center Berlin Germany

**Keywords:** autophagy‐derived secretome, EV secretion, intercellular communication, secretory autophagy, unconventional secretion, Autophagy & Cell Death, Membranes & Trafficking, Signal Transduction

## Abstract

Over the recent years, it has become apparent that a deeper understanding of cell‐to‐cell and organ‐to‐organ communication is necessary to fully comprehend both homeostatic and pathological states. Autophagy is indispensable for cellular development, function, and homeostasis. A crucial aspect is that autophagy can also mediate these processes through its secretory role. The autophagy‐derived secretome relays its extracellular signals in the form of nutrients, proteins, mitochondria, and extracellular vesicles. These crosstalk mediators functionally shape cell fate decisions, tissue microenvironment and systemic physiology. The diversity of the secreted cargo elicits an equally diverse type of responses, which span over metabolic, inflammatory, and structural adaptations in disease and homeostasis. We review here the emerging role of the autophagy‐derived secretome in the communication between different cell types and organs and discuss the mechanisms involved.

## Graphical abstract

Autophagy is indispensable for cellular development, function, and homeostasis and mediates these processes through its secretome. This review discusses roles of the autophagy‐derived secretome in the communication between cells and organs and the mechanisms involved.

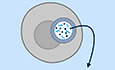

## Overview of autophagy

Autophagy is an evolutionary highly conserved cellular degradation and recycling process, indispensable for cellular and organ development, homeostasis, and function (Dikic & Elazar, 2018). It serves as a quality control mechanism for removal of damaged or dysfunctional organelles or large protein aggregates. In addition, it is an energy‐generating pathway that recycles cytoplasmic macromolecules to replenish energy and anabolic precursor pool during starvation. Through these functions, autophagy acts as a master regulator of cellular metabolism (Rabinowitz & White, 2010; Deretic & Kroemer, 2022). Furthermore, autophagy is also implicated in effector functions of the immune response through regulation of cellular metabolic state, survival, and antigen presentation (Clarke & Simon, 2018). Cellular and environmental stress, nutrient and energy availability, growth factor as well as hormone sensing all regulate autophagy activation.

There are many excellent reviews on the mechanistic details of degradative autophagy and the reader is referred to them (Levine & Klionsky, 2004; Parzych & Klionsky, 2014; Dikic & Elazar, 2018). In brief, the most prevalent autophagic process—macroautophagy—can be summarized in four main steps. First, the autophagosome formation is initiated through the mTORC1 and AMPK‐mediated activation of unc51‐like autophagy‐activating kinase 1 (ULK1) complex. Second, the formation and elongation of the nascent autophagosome is facilitated by several ATG factors, which specify the site of lipidation and mediate the conjugation of phosphatidylethanolamine (PE) to autophagosome‐bound LC3. Third, this membrane‐bound LC3 directs the growth, closure and maturation of the autophagosome. Finally, a mature autophagosome fuses with a lysosome which results in acid hydrolase‐mediated degradation and recycling of autophagic cargo that is recycled in forms of molecular building blocks such as amino acids, glucose, and lipids (Glick *et al*, 2010; Feng *et al*, 2014).

Besides macroautophagy, different types of degradative autophagy have been studied to date, including microautophagy, chaperone‐mediated autophagy, non‐canonical autophagy as well as selective autophagy, which is substrate‐specific; examples are mitophagy for mitochondrial clearance and lipophagy for degradation of lipid droplets (Singh & Cuervo, 2012; Boya *et al*, 2013; Cuervo & Wong, 2014; Onishi *et al*, 2021). In contrast to these types of autophagy, secretory autophagy has a role in facilitating unconventional cellular secretion of biological cargo for the communication between cells (Nguyen & Debnath, 2022).

In this review, we focus on the functional aspects of secretory autophagy in facilitating cell‐to‐cell and organ‐to‐organ communication. We explore how these autophagy‐mediated networks function in metabolic, inflammatory, and structural adaptations, spanning both local and systemic environments. Furthermore, we summarize the extracellular signals that are relayed through non‐cell autonomous autophagy that act to functionally modulate recipient cells and organs in homeostasis and disease.

## Secretory autophagy at the crossroads of endo‐ and exocytosis

Secretory or non‐cell autonomous autophagy is an umbrella term for autophagy‐mediated extracellular secretion through autophagosomes/endosomes, including conventional and unconventional secretion of proteins, extracellular vesicle (EV) production, and egress of secretory lysosomes (Leidal *et al*, 2020). The pathway appears to be triggered upon cell starvation, ER stress, unfolded protein response (UPR), and in the event of intracellular trafficking blockade (Jahangiri *et al*, 2022).

**Figure 1:**
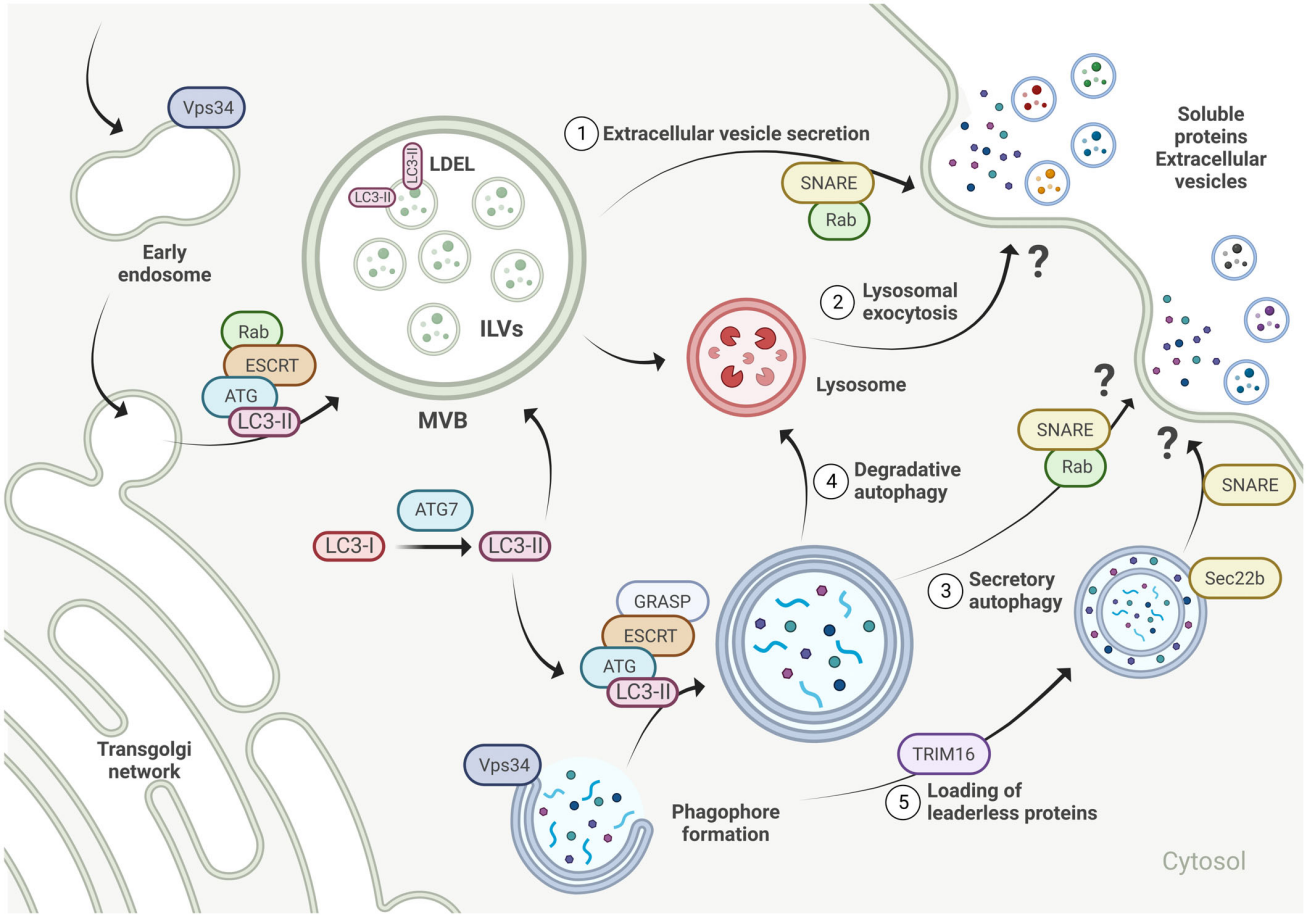
Secretory autophagy is at the crossroads of endo‐ and exocytosis Secretory autophagy (3, 5) overlaps with endo/exocytotic pathways (1) and lysosomal exocytosis (2) and the key overlapping factors are highlighted here. Branching away from degradative autophagy (4), there is still some inconsistency in the current reports on the exact mechanism of the secretory pathway, therefore we have often highlighted protein families, rather than individual proteins. Furthermore, the mechanism of fusion of autophagosome with the plasma membrane remains elusive. See the main text for a detailed description of the pathway. The figure was created with BioRender.com.

Indeed, growing evidence suggests a close overlap of secretory autophagy and endo/exocytotic pathways (Deretic *et al*, 2012; Fig 1). Endocytosis mediates intracellular trafficking of cargo into the cell via intraluminal vesicles (ILVs) of late endosomes/multivesicular bodies (MVBs) that eventually fuse with the lysosome. Notably, several endosomal regulators are also critical for autophagy initiation, maturation, and fusion with the lysosome, including Vps34, ESCRT, and ATG proteins (Fig 1). In addition, autophagosomes share their membrane origin with endosomes (Papandreou & Tavernarakis, 2020). Likewise, in exocytosis late endosomes/MVBs fuse with the cell membrane to release ILVs in form of exosomes and EVs into the extracellular environment (Jahangiri *et al*, 2022; Nguyen & Debnath, 2022). The biogenesis and release of autophagic, intracellular, and extracellular vesicles involve a significant number of common regulators, including LC3, SNARE, Rab, and ESCRT family proteins (Fig 1), as well as post‐translational modifications critical for cargo sorting (Gudbergsson & Johnsen, 2019; Xing *et al*, 2021). Due to these parallel mechanisms, secretory autophagy is often thought of as a compensatory mechanism of dysfunctional endo‐, exocytosis, or EV biogenesis observed in numerous human diseases including cancer, diabetes mellitus, neurodegenerative, and cardiovascular diseases (Yarwood *et al*, 2020).

Notably, it has not always been clearly shown in secretory autophagy studies whether a loss of autophagy actually leads to a loss of intracellular vesicles resembling autophagosomes or whether autophagy is required to provide the necessary energy or molecules to form these vesicles. Currently, the most attractive hypothesis is that the double‐membraned structure of the autophagosome follows the exocytotic pathway where the outer membrane fuses with the plasma membrane and the inner membrane forms the secretory vesicle. Understanding the molecular intricacies of secretory autophagy is still in its infancy, with many open questions, however, several landmark studies have recently shed light on the nature of secreted cargo and a promising role of autophagy in intercellular and interorgan communication in diverse physiological and pathological processes, which we will highlight below.

## Autophagy‐derived messengers

**Figure 2:**
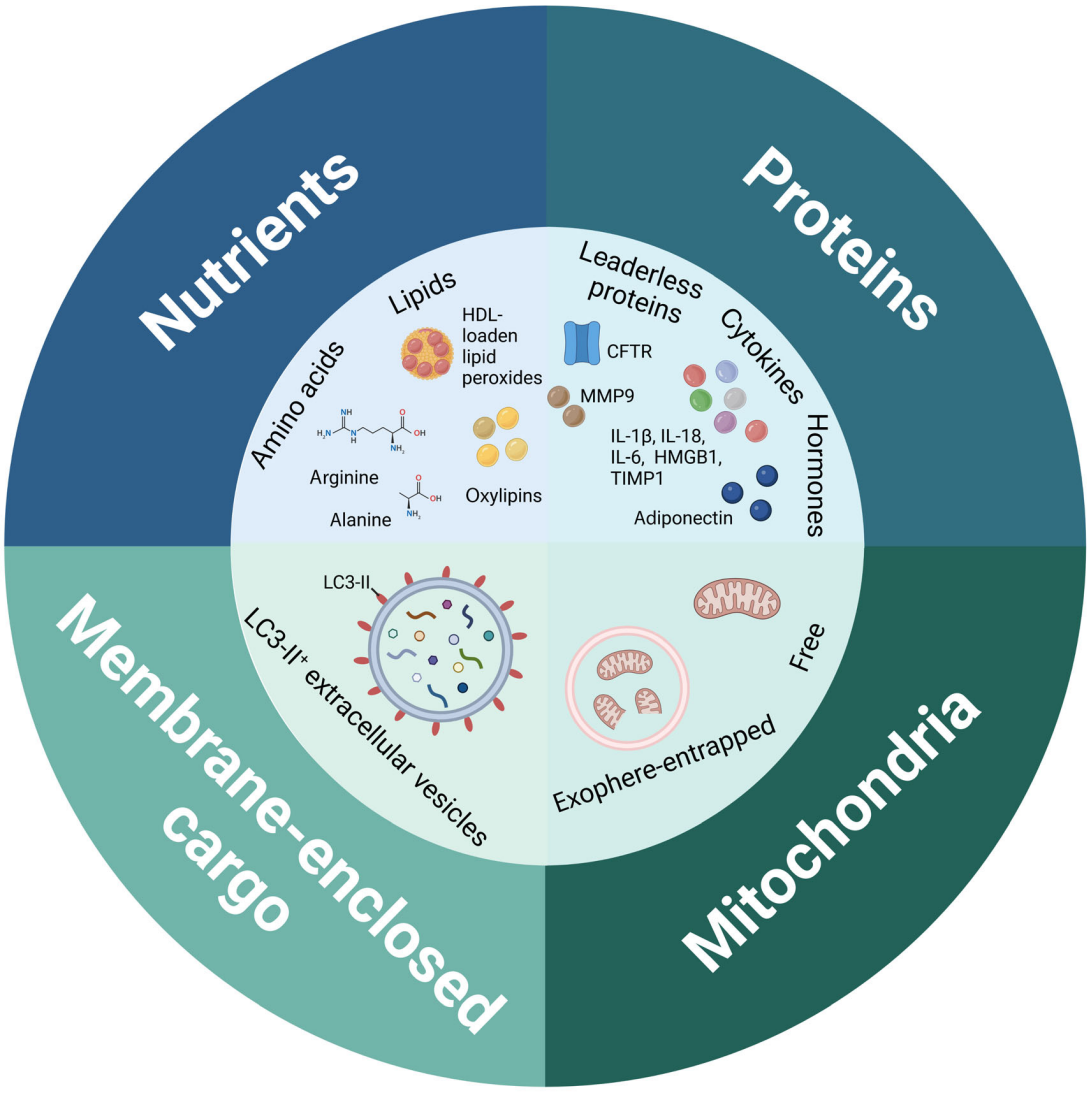
The autophagy‐derived secretome The diverse nature of secreted autophagy cargo includes amino acids, lipids, proteins, mitochondria, and membrane‐bound cargo. The molecules and mechanisms of secretion are described in detail in the main text. The figure was created with BioRender.com.

Although traditionally seen as a means of waste material extrusion from a cell, it has now become apparent that the secretory autophagy cargo comprises functional messengers. The nature of the autophagy‐derived secretome can be diverse, including amino acids, lipids, proteins, mitochondria, and membrane‐bound cargo (Fig 2). Understanding autophagy‐derived messengers is critical to broaden our understanding of the mechanisms involved and might provide interesting avenues for future therapeutic interventions targeting autophagy.

### Nutrients

Secretion of nutrients, such as amino acids and lipids, that are either a result of catabolism of intracellular proteins or enzymatic conversion, has been related to the main recycling function of autophagy (Fig 2). Secreted amino acids, such as arginine and alanine, and adenosine triphosphate (ATP), impact extracellular metabolic interactions and survival, as reported in tumors across different species (Martins *et al*, 2014; Sousa *et al*, 2016; Katheder *et al*, 2017; Poillet‐Perez *et al*, 2018).

Besides amino acids, secretion of lipid species such as lipid peroxides and oxylipins has also been reported to be autophagy‐dependent, with secretory autophagy assisting in lipid loading onto lipoproteins or lipid hydrolysis (Cai *et al*, 2018; Richter *et al*, 2023).

### Proteins

Secretory autophagy has been implicated in non‐degradative secretory pathways of bioactive proteins or residual proteins both in yeast and in mammals (Fig 2). Here many studies include detailed molecular mechanisms of secretion.

The first studies of autophagy machinery's role in the unconventional secretion of leaderless proteins were done in yeast. Two groups independently demonstrated that secretion of the protein Acb1 requires the autophagy machinery (ATG5, ATG7, ATG8, and ATG12) and Golgi‐associated GRASP protein Grh1. Under starvation, Acb1‐containing autophagosomes evade degradation by fusing with recycling endosomes instead of the vacuole, forming MVBs that will release the protein after delivery to the plasma membrane. Importantly, these studies did not demonstrate the mechanism by which specific cargo was sorted into the autophagosomes (Duran *et al*, 2010; Manjithaya *et al*, 2010).

Similar to the yeast studies, the secretion of integral membrane protein CFTR was also shown to depend on an unconventional route via the autophagosomal machinery (ATG1, ATG5, ATG7, and ATG8) and GRASP. Importantly, the unconventional surface trafficking is physiologically relevant in cystic fibrosis as it can promote ΔF508‐CFTR misfolded mutant protein delivery to the membrane. This enforces its channel function and alleviates the disease, suggesting that targeting secretory autophagy might serve as a potential therapeutic strategy for management of misfolded proteins (Gee *et al*, 2011).

Dupont *et al* were the first ones to show the biosynthetic role of autophagy in the unconventional secretion of the cytokines interleukin 1β and 18 (IL‐1β and IL‐18) in mammals, both members of the IL‐1 family. Upon inflammasome activation, these proinflammatory substrates are transported in autophagic vacuoles from the cytosol to the plasma membrane, followed by exocytosis. This facilitates a cytoplasmic exit for molecules without leader peptides that would conventionally be trafficked through the ER. The authors unraveled that besides ATG5 of the autophagic machinery, the export depends on the Golgi‐associated protein GRASP55, required for autophagy initiation as well as on the exocytic vesicular transport regulator Rab8a which was found to colocalize with IL‐1β + autophagosomes. Notably, the extracellular delivery of these proinflammatory cytokines was promoted by starvation, while inhibited under fed, basal autophagy condition (Nakahira *et al*, 2011; Zhou *et al*, 2011). Autophagy‐mediated secretion of IL‐1β was also observed in human neutrophils, which are known to be an important source of this cytokine during bacterial and fungal infection (Iula *et al*, 2018). Interleukin 1β findings were further expanded to secretion of alarmin HMGB1, which highlights the role of autophagy in unconventional secretion of multiple inflammasome‐related factors and suggests that this autophagic pathway might couple metabolism and inflammation in idiopathic inflammatory and infectious diseases (Thorburn *et al*, 2008; Dupont *et al*, 2011; Kim *et al*, 2021).

While Dupont *et al* clearly demonstrated that autophagy plays a role in the secretion of mature IL‐1β, the molecular underpinnings of the protein export remained unexplained. Several groups have since used IL‐1β as a model system to study mechanistic details of autophagy‐dependent secretion of leaderless proteins. In contrast to the non‐specific engulfment of cytosolic material that is destined for degradation, the loading of mature IL‐1β into LC3‐II^+^ vesicles was observed to be mediated by a direct interaction with HSP90. Furthermore, the authors demonstrated that the entry of IL‐1β into these autophagosome‐precursor vesicles is critical to localize the protein between the outer and inner membrane of autophagosome double membrane, aiding to its secretion as a soluble form (Zhang *et al*, 2015). Their work was further expanded in a more recent study offering a better understanding of the IL‐1β translocation pathway. TMED10 was identified as another critical factor directly facilitating the IL‐1β translocation into the vesicle by oligomerization‐dependent protein channel formation (Zhang *et al*, 2020). While Zhang *et al* concluded that IL‐1β is secreted as a soluble protein either via autophagosome‐plasma membrane direct fusion or via the MVB pathway, Kimura *et al* explored the mechanistic details of the export itself in depth, identifying dedicated secretory autophagy cargo receptor and SNARE proteins. Leaderless cargo is recruited to LC3‐II^+^ vesicles with the help of receptor TRIM16 which interacts with a specific R‐SNARE Sec22b protein to load the IL‐1β into the autophagosome precursor and secrete the cargo through interaction with plasma membrane Qa‐SNAREs syntaxin 3 and syntaxin 4 (Kimura *et al*, 2017). While there are some disparities between the factors involved, these mechanistic findings could potentially apply to other leaderless cargoes, including ferritin (Kimura *et al*, 2017).

Autophagy (ATG7 and ATG12) is also believed to facilitate production and conventional secretion of the cytokine IL‐6, which drives oncogenic RAS invasion of epithelial cells and pulmonary metastasis *in vivo*. Albeit authors claim that autophagy promotes cancer progression through secretion of several factors, including IL‐6, these claims are not molecularly defined, and more evidence is required to understand the role of autophagy in this unconventional secretion (Lock *et al*, 2014).

Further autophagosome secretome proteins were found in a screen by Kraya *et al*, who aimed at identifying candidate autophagy biomarkers that would predict the success of therapeutic autophagy modulation. To this end, they compared the secretome of low‐autophagy melanoma cells and their high‐autophagy metastatic derivative, and confirmed their observations in serum of metastatic melanoma patients, identifying IL‐1β, CXCL8 (chemokine (C‐X‐C motif) ligand 8), LIF (leukemia inhibitory factor), FAM3C (family with sequence similarity 3, member C), and DKK3 (dickkopf WNT signaling pathway inhibitor 3). Nevertheless, further research of the link between autophagy‐mediated unconventional secretion and secretion of these factors is necessary to understand the molecular underpinnings of the process (Kraya *et al*, 2015).

Glucocorticoid‐mediated cellular stress facilitates the secretion of matrix metalloproteinase 9 (MMP9) from microglia in the medial prefrontal cortex via secretory autophagy. Extracellular secretion of MMP9 via FK506‐binding protein 51 leads to enhanced maturation of brain‐derived neurotrophic factor, promoting synaptic plasticity *ex vivo*. This can in turn lead to neuroinflammation and depression, suggesting that targeting secretory autophagy in the nervous system might be beneficial for stress‐related disorders (Martinelli *et al*, 2021).

Importantly, hormones can also be an autophagy cargo. An example of autophagy‐mediated hormone secretion was reported by Kuramoto *et al*, who identified adiponectin as being secreted with the help of the autophagy machinery factor Becn1. The study showed that Becn1 directly binds a Sec6 component of a vesicle trafficking multiprotein complex exocyst, which facilitates adiponectin secretory vesicle docking and secretion from the plasma membrane. Notably, the Becn1‐exocyst interaction is distinct from the unconventional secretion of IL‐1β and IL‐18, which depends on autophagosome‐like vesicles (Kuramoto *et al*, 2021).

Altogether these discoveries demonstrate that autophagy is indispensable for secretion of nutrients and bioactive molecules. Understanding these autophagy‐driven dependencies and cooperations will be critical to better target systemic diseases with autophagy‐modulating drugs.

### Mitochondria

Secretory autophagy also plays a role in the elimination of mitochondria from cells (Fig 2). The process, termed autophagic secretion of mitochondria, was reported to occur as a response to defects in the mATG8‐conjugation machinery and is lysosome‐independent. Instead, the autophagosome is redirected towards the plasma membrane, acting itself as a vesicular carrier for secretory cargoes, however, the mechanistic details of this unconventional secretion remain unclear. The switch from degradative to secretory autophagy is critical for removal of dysfunctional or damaged mitochondria and is part of mitochondrial quality control. The extruded mitochondria activate the innate immune cGAS‐STING pathway and enhance the secretion of pro‐inflammatory cytokines, suggesting that functional autophagy, in particular mATG8 lipidation is critical in suppressing inflammatory responses (Tan *et al*, 2022). Similar mechanism of action could contribute to the observations made by Davis *et al* (2014), who observed transcellular degradation of mitochondria, called transmitophagy in the CNS.

In contrast to these studies, cellular mitochondrial clearance has also been described to depend on vesicle entrapment. Exopheres, membrane‐surrounded vesicles, were found to mediate secretion of damaged mitochondria from cardiomyocytes (Fig 2). Their biogenesis was autophagy dependent, shown by an overlap with LC3^+^ positive puncta, together with their secretion being positively regulated via rapamycin treatment (mTOR inhibition) or negatively by genetic block of autophagy ATG7 deletion (Nicolás‐Ávila *et al*, 2020).

### Membrane‐enclosed cargo

The first hint for the involvement of autophagy machinery in the secretion of membrane‐bound cargo became apparent in 2015, when ATG12 and ATG3 were reported to interact with the ESCRT‐associated protein Alix, thereby controlling late endosome biogenesis and function (Murrow *et al*, 2015). Leidal *et al* were the first to describe a direct role of LC3 and the LC3‐conjugation machinery in the secretion of membrane‐bound cargo (Fig 2). The authors describe a distinct and unconventional function of the autophagic machinery in protein and RNA cargo loading into specific EVs for secretion. In this process, termed LC3‐dependent EV loading and secretion (LDELS), lipidated LC3‐II at the MVB membrane specifically captures the cargo and incorporates it into ILVs for EV‐dependent release upon MVB fusion with the plasma membrane. Importantly, this pathway relies on LC3 localized at single membrane endosomes rather than double membrane autophagosomes. In contrast to initial observations by Murrow *et al*, here the secretion is largely independent of the ESCRT machinery. Using proximity‐dependent proteomics (Box 1), the authors identified RNA‐binding proteins, including HNRNPK and SAFB, small non‐coding RNAs and EV cargoes as targets that undergo loading and secretion via the LDELS pathway. The impairment of LDELS via ATG7 and ATG12 deletion resulted in a profound dysregulation of extracellular small non‐coding RNAs, suggesting the importance of the pathway in regulating proteostasis via post‐transcriptional control during cellular stress (Leidal *et al*, 2020). These observations were further expanded by the finding that extracellular secretion of the transferrin receptor depends on LDELS, whereby the LC3‐conjugation machinery directly binds the receptor's cytosolic domain and thus specifies membrane protein loading onto EVs for secretion (Gardner *et al*, 2022).

Box 1 **Methods to study autophagy‐mediated intercellular and interorgan crosstalk**

An important piece of the puzzle in understanding non‐cell autonomous functions of autophagy is identifying the cargoes that are directed towards the autophagosomes. To identify the individual proteins and protein complexes that are engulfed by autophagosomes, a proximity labeling‐based method can be utilized, where engineered APEX2 peroxidase is genetically targeted to autophagosomes via fusion with hATG8/LC3 protein (Le Guerroué *et al*, 2017; Leidal *et al*, 2020). This spatial restriction enables enzymatic tagging of nearby endogenous proteins by addition of a substrate, and labeled proteins can be profiled using mass spectrometry‐based quantitative proteomics (Hung *et al*, 2016). Proximity labelling‐based proteomics allows the identification of novel candidate cargo proteins, the study of the dynamics of autophagosomal content across different biological material (cell lines, primary cells, whole organism) and growth conditions, as well as the understanding of the functional relevance of autophagy in homeostasis and disease. Recently, a new method for isolation of native autophagic vesicles using ATG8 antibodies‐based FACS (fluorescence‐activated cell sorting) has been described. This method, in contrast to other cellular‐fractionation‐based and proximity‐based methods, allows for isolation of autophagic vesicles in large quantities with minimal cell disruption and does not require genetic manipulation. FACS‐purified intact autophagic vesicles are suitable for lipid and protein cargo profiling (Schmitt *et al*, 2022). Similarly, intact LC3‐positive autophagic vesicles can be isolated in a two‐step process combining a gradient‐dependent cell fractionation for autophagic vesicle enrichment and immunoprecipitation of LC3‐positive autophagic vesicles (Kallergi *et al*, 2023).

The involvement of autophagy machinery in EV secretion was further strengthened by another study from the same group. This study demonstrated increased EV secretion upon endolysosomal dysfunction or impaired autophagosome formation/maturation by bafilomycin A1 or chloroquine, and these vesicles contained autophagic cargo receptors, including p62. Based on this, the authors proposed that vesicle‐based secretory autophagy acts as an alternative pathway to maintaining intracellular protein homeostasis by degradative autophagy when the latter is impaired (Solvik *et al*, 2022).

Similar observations were made by Xu *et al*, who also demonstrated that lysosomal inhibition by chloroquine alters the cellular secretome and induces the release of small EVs. These originate from a double‐membrane source and are rich in Atg8 orthologs and autophagy cargo receptors, similar to the results by Solvik *et al*. Notably, together these findings indicate that autophagy machinery contributes to the heterogeneity and composition of small EVs (Xu *et al*, 2022).

These observations have recently been validated in B cells, where B‐cell activation through external IL‐4:CD40 co‐stimulation enhanced autophagosome‐like EV secretion. As in previous studies, release of EVs was facilitated by a reduced autophagosome–lysosome fusion and RAB27a via NF‐κB2 signaling pathway. RAB27a was proposed to promote the EV secretion by directly guiding autophagosome docking with the plasma membrane, followed by either exosome secretion or membrane shedding. As the authors observed circulating B‐cell‐derived LC3‐II^+^ EVs in blood stream during homeostasis and lymphoma formation, they proposed that this mechanism serves to establish protective immune responses and/or promote tumorigenesis. Nevertheless, these hypotheses remain to be experimentally proven (Kuan *et al*, 2023).

In summary, expanding our understanding of the autophagy‐derived secretome represents an important venue for future research. Other factors have been proposed to play a role in autophagy‐mediated secretion of membrane‐enclosed cargo, including the BORC‐ARL8‐HOPS pathway, which has recently been identified as a decision maker between the rate of endolysosomal fusion and exosome secretion (Shelke *et al*, 2023). Besides mediating intercellular communication, a potentially important application of autophagy‐derived extracellular molecules lies in their use as biomarkers in liquid biopsies, as it has been shown for EVs (Gudbergsson & Johnsen, 2019).

## Autophagy in the control of cell‐to‐cell communication

**Figure 3:**
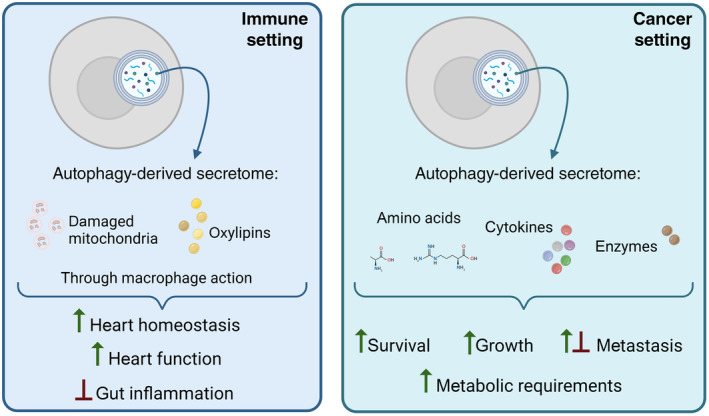
The role of secretory autophagy in the crosstalk between cells Cell types communicate their metabolic needs and/or extent of damage to neighboring cells, often of different origin. This figure highlights the role of the autophagy‐derived secretome in maintenance of some of these intricate networks. Detailed mechanisms of these interactions are discussed in the main text. The figure was created with BioRender.com.

Cells communicate their nutrient and energy requirements or pathological defects through secretion of mediators, which signal to other cells within the tissue to maintain tissue homeostasis. This crosstalk often happens between cells of different types, suggesting that the interaction is based on a task allocation between functionally specialized cell types. The functionality of these cellular networks is supported by secretory autophagy and several examples are described below (Fig 3).

### Host and cancer cells in mediating cancer pathogenesis

Interaction between host and cancer cells is an evolutionary‐conserved autophagy‐mediated cellular crosstalk described in *Drosophila melanogaster*, mouse, and human cell lines. Cancer cells are often stressed due to accumulation of damaged mitochondria and ROS and additionally have an increased metabolic demand to sustain their growth and proliferation. They can meet this demand by increasing the import of nutrients from their microenvironment and alternating metabolic fuel sources. Autophagy represents an important nutrient source both within the cell and in its microenvironment, and as such metabolically controls tumor growth.

The importance of an autophagy‐mediated crosstalk between host and cancer cells was first described by Sousa *et al* in pancreatic ductal adenocarcinoma (PDAC), where the upregulation of autophagy is indispensable for tumor growth (Yang *et al*, 2011). The authors reported that stroma‐associated pancreatic stellate cells (PSCs) promote PDAC tumor initiation through autophagy‐mediated protein degradation. They showed that cell‐extrinsic autophagy acts as a source of secreted alanine, which is utilized by PDAC cells as an alternative carbon source to fuel biosynthesis reactions. Notably, the crosstalk appears to be two‐way, since the increase in stromal autophagy is signaled by the PDAC cells, and a genetic inhibition of autophagy in PSCs results in altered metabolism that could be rescued by the addition of exogenous alanine. This metabolic support offers the tumor a shift in its primary fuel source, reducing its dependence on limited nutrients in the microenvironment and promoting its growth (Sousa *et al*, 2016).

The work from Sousa *et al* was further expanded by Yang *et al*, who aimed at understanding the difference between cell‐autonomous and non‐cell‐autonomous autophagy in support of PDAC growth. Using a mouse model with acute and reversible inhibition of autophagy, they demonstrated that the inhibition of whole‐body host autophagy contributes to PDAC treatment through a delayed tumor formation, while tumor maintenance is supported via tumor cell‐intrinsic autophagy. These observations endorse the inhibition of autophagy as a therapeutic target in PDAC and highlight the complexity and differential roles of cell‐intrinsic and cell‐extrinsic roles of autophagy in tumor growth (Yang *et al*, 2018).

Katheder *et al* were the first to report that autophagy is indispensable for tumor growth in both the local tumor microenvironment as well as in distant somatic tissues, including muscle, gut, and fat body, and tumor expansive invasion in *Drosophila melanogaster*. They demonstrated that metabolically stressed tumor cells induce non‐cell autonomous autophagy through tumor necrosis factor and interleukin‐6‐like signaling to support their own growth. Local provision of recycled nutrients, such as amino acids, through genetic and pharmacological inhibition of autophagy results in a restrained early‐stage tumor proliferation and dissemination into neighboring tissue (Katheder *et al*, 2017).

Another example of how the host autophagy supports specific metabolic requirements of tumor growth was described by Poillet‐Perez *et al*, who report a role of autophagy in arginine dependency of multiple allografted tumors. The growth of arginine‐auxotrophic tumors, including melanoma, urothelial carcinoma, and non‐small‐cell lung cancer is impaired in the absence of host autophagy due to liver damage, which leads to release of arginase I and subsequently depletion of circulating arginine. As reported previously (Sousa *et al*, 2016; Katheder *et al*, 2017), they demonstrated that autophagy provides amino acids in the tumor microenvironment, through either local or systemic action, supporting tumor metabolic demands. At the same time, they highlighted differential dependencies of tumors on host autophagy, further emphasizing the need of better mechanistic understanding to develop targeted therapies (Poillet‐Perez *et al*, 2018).

A similar indirect role of autophagy in the release of tumorigenesis factors has been reported in relation to chemotherapy‐induced immunogenic cell death. Autophagy has been demonstrated as required—but not sufficient—for release and maintenance of extracellular ATP levels in the tumor microenvironment, thereby promoting therapeutic antitumor immune response. The authors observed that in the autophagy‐deficient colorectal carcinoma tumors, the release of the ATP chemotactic signal from dying tumor cells was suppressed. In turn, these tumors failed to recruit myeloid cells and prime the immune response upon chemotherapy and were hence less responsive to treatment (Michaud *et al*, 2011). While the process was rigorously shown to rely on the induction of premortem autophagy, the authors later demonstrated that rather for secretion itself, autophagy is responsible for maintenance and availability of the lysosomal ATP pool, and that secretion depends on lysosomal exocytosis (Wang *et al*, 2013; Martins *et al*, 2014).

Besides metabolic control, autophagy also plays a role in promoting the secretion of tissue inhibitor of metalloproteinase 1 (TIMP1), an inflammatory cytokine. As suggested by the authors, the release of TIMP1 by secretory autophagy is observed upon starvation and depends on Rab37‐mediated exocytosis. The authors suggested that the starvation‐induced autophagy serves as an upstream signal for activation of Rab37, and identified LC3‐anchored vesicles, p62, and Sec22b as the participatory autophagy machinery. The secreted TIMP1 suppresses the motility of lung cancer cells *in vitro* and *in vivo*, contributing to decreased metastasis and tumor nodules (Wu *et al*, 2022).

In summary, these studies highlight the role of autophagy in the metabolic and cytokine crosstalk between tumor cells and its microenvironment by exploring tumor‐specific metabolic vulnerabilities and adaptation mechanisms. Host autophagy supports the tumor microenvironment though both local as well as systemic effects, in turn mediating metabolism, proliferation, survival, and malignancy of tumors (Fig 3). Anti‐cancer therapies targeting autophagy are already being tested, however, a deeper understanding of cancer cell dependence on cell‐autonomous versus secretory autophagy is necessary to better restrain tumor growth and invasion. Additionally, it is important to understand the complexity of targeting autophagy systemically since it can potentially lead to decreased immune surveillance.

### Immune and non‐immune cells in mediating immunity

Cells of the immune system can also alter their metabolic demands in response to different stimuli and are dynamic in doing so.

Non‐cell autonomous autophagy not only mediates metabolic, but also immune crosstalk within the tumor microenvironment. In contrast to low tumor mutational burden (TMB) tumors described in the previous section, host autophagy supports the survival of high‐TMB tumors through immune rather than metabolic mechanisms. This is achieved through a sustained regulatory T‐cell function, T‐cell exhaustion, and downregulation of proinflammatory components. Specifically, host autophagy suppresses the STING pathway, leading to limited IFN type I and II responses and antigen presentation, supporting tumor growth. This tumor immune tolerance has been observed to depend on liver host autophagy and has been therefore termed hepatic autophagy immune tolerance (Poillet‐Perez *et al*, 2020).

Besides orchestrating tumor‐specific immune vulnerabilities, secretory autophagy is also critically shaping immune responses in other pathological conditions. In their study, Nicolás‐Ávila *et al* discovered a critical role of autophagy in preserving proteostasis and heart function through ejection of vesicles called exophers. This housekeeping function is further enhanced during cardiac stress and is responsible for a discharge of dysfunctional mitochondria into extracellular space, where these subcellular particles are phagocytosed by cardiac‐resident macrophages and eliminated. Blocking this “outsourcing” process by macrophage depletion results in the activation of the inflammasome, block of autophagy, and heart dysfunction. This study thus highlights an autophagy‐driven non‐canonical crosstalk between parenchymal and immune cells, which is indispensable for the support of heart homeostasis and function. Functionally, this study clearly demonstrates that autophagy is not solely important for the metabolic crosstalk between the cells in forms of nutrient exchange but is also critical for the exchange of other materials, such as mitochondria (Nicolás‐Ávila *et al*, 2020). In addition, the findings of this paper confirm previous observations, where a non‐cell autonomous autophagy was utilized to fuel metabolic demands of cells with dysfunctional mitochondria (Katheder *et al*, 2017), although the molecular mechanisms appear distinct (autophagy‐mediated nutrient exchange versus elimination). Autophagy‐mediated transcellular degradation of damaged mitochondria has also been predicted to occur in the adipocyte–macrophage crosstalk however, this hypothesis remains to be confirmed (Crewe *et al*, 2021).

**Figure 4:**
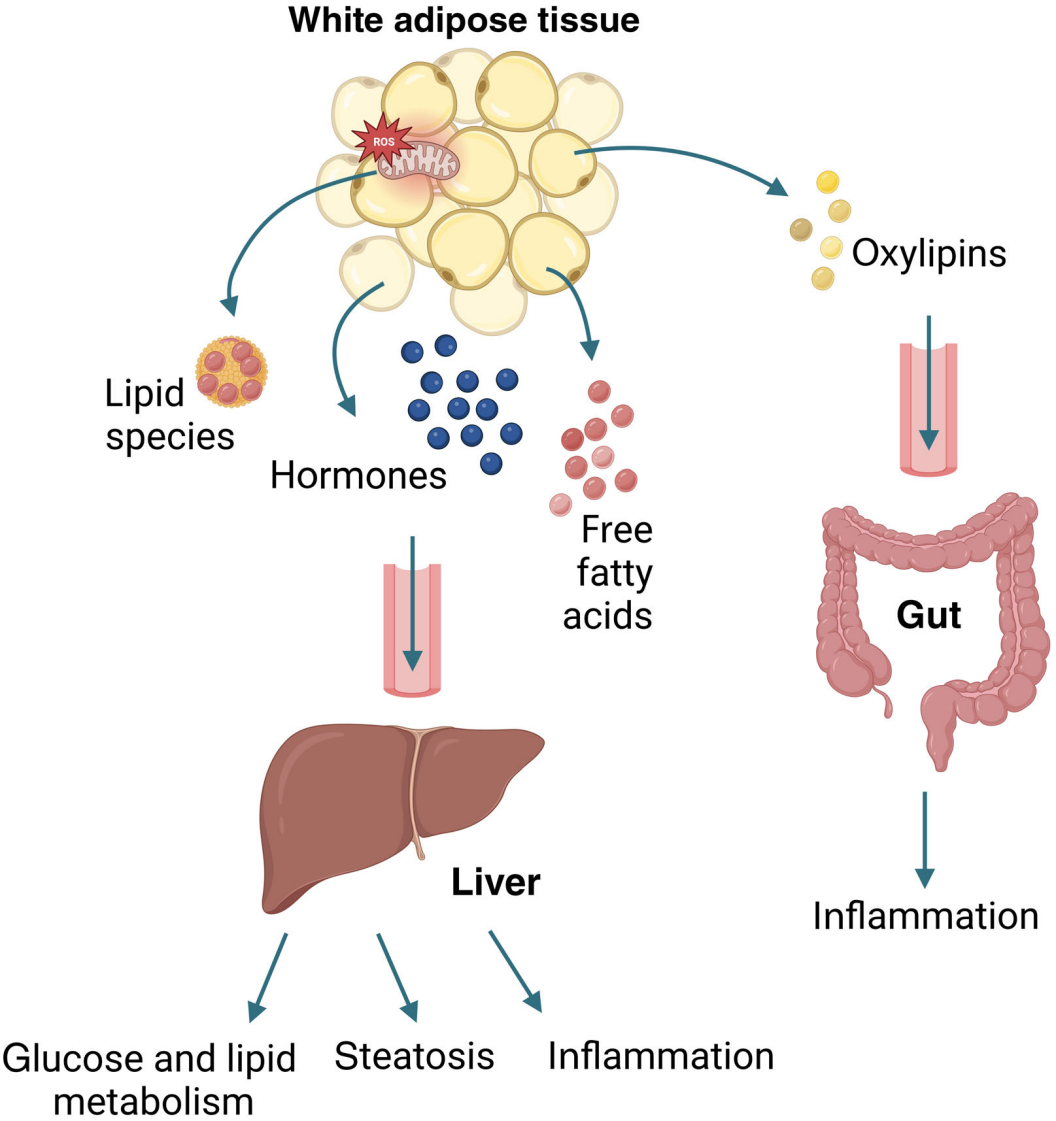
The role of secretory autophagy in the crosstalk between adipose tissue and liver or gut Adipose tissue communicates its metabolic needs and/or extent of damage to distinct sites, such as liver and gut. This figure highlights the role of adipose tissue autophagy‐derived functional molecules and their downstream effects. Detailed mechanisms of these interactions can be found in the main text. The figure was created with BioRender.com.

Another study underlies the importance of non‐cell‐autonomous autophagy in the mediation of inflammatory responses. Richter *et al* demonstrated that autophagy in adipocytes limits DSS‐induced intestinal inflammation by controlling enzymatic lipid oxidation in adipose tissue. The secreted oxygenated polyunsaturated fatty acids, called oxylipins, in turn control anti‐inflammatory IL‐10 cytokine production from adipose tissue macrophages. After entering the circulation, adipose tissue‐derived IL‐10 mediates distant inflammation in the gut, implicating the role of autophagy in the local adipocyte–immune system and systemic adipose tissue–gut crosstalk during injury‐induced gut inflammation in mice (Fig 4). Similar observations were made in Crohn's disease patients, implicating a therapeutic value of targeting the mentioned crosstalk (Richter *et al*, 2023).

In summary, autophagy appears to be indispensable in a crosstalk between immune and non‐immune cells, not only in disease but also in maintenance of homeostasis (Fig 3). Furthermore, studies of cell‐extrinsic roles of autophagy in relation to immune system reveal that autophagy not only plays a role in amino acid exchange as reported in cancer–host interactions but also in vesicular and lipid exchange. Notably, studies investigating the role of non‐cell autonomous autophagy in relation to the immune system mostly focus on highly specialized cells that have substantial metabolic demands and a low turnover, strengthening the idea that this process serves as an adaptive mechanism to cellular stress and damage.

## Autophagy in the control of organ‐to‐organ communication

Autophagy‐derived messages are not only exchanged between cells of the same tissue, for example, within the pancreas (Sousa *et al*, 2016), heart (Nicolás‐Ávila *et al*, 2020), or other tissue microenvironments (Katheder *et al*, 2017), but also systemically between distant tissues, regulating whole‐body energy balance, metabolism, and function (Fig 4).

Regulation of glucose homeostasis and peripheral insulin sensitivity occurs through interplay of liver, adipose tissue, and skeletal muscle. The role of autophagy within cells that constitute these tissues has been well studied (Ueno & Komatsu, 2017; Romero & Zorzano, 2019; Frendo‐Cumbo *et al*, 2021), however, understanding how the function of autophagy could span beyond those tissues as well as being involved in inter‐tissue communication has only recently started to emerge. Several authors explored the role of adipocyte autophagy in the maintenance of whole‐body and tissue homeostasis. Adipose tissue is an important endocrine organ and adipocytes are responsible for lipid storage, regulating the energy metabolism of the body (Berg & Scherer, 2005). Therefore, it is no surprise that most studies looked at adipose tissue autophagy and how this could play a role in the communication with other metabolic tissues.

By studying autophagy in mature adipocytes, Cai *et al* discovered its cell‐extrinsic role, responsible for mediating a whole‐body peripheral insulin sensitivity and glucose homeostasis, independent of diet or adiposity. The authors first showed that the lack of autophagy results in a cell‐intrinsic defect, that is, accumulation of dysfunctional mitochondria with impaired mitochondrial morphology and function in adipocytes. This leads to elevated lipid peroxide formation and Nrf2 and Keap1 signaling, suggesting a role of autophagy in alleviating oxidative injury in the adipocytes. Notably, depletion of adipocyte autophagy uncovered an adipose‐liver crosstalk, as it resulted in the transfer of lipid peroxides from adipocytes to insulin‐sensitive peripheral tissues, including liver and muscle. In the liver, this led to upregulation of Nrf2 and Keap1 signaling and increased hepatic gluconeogenesis, indicating that autophagy is an indispensable signaling mechanism for adipose tissue to convey its oxidative damage to liver, thereby promoting its detoxification function and metabolism. Strikingly, the systemic metabolic impairments caused by the absence of autophagy in adipocytes might be contributing to diabetes and cardiovascular disease (Cai *et al*, 2018).

Autophagy‐mediated fat‐liver crosstalk was further explored by Sakane *et al*, who demonstrated that white adipose tissue autophagy exacerbates liver steatosis, inflammation, and fibrosis. The study proposes that modulation of adipocyte autophagy may represent an effective treatment for NAFLD/NASH through attenuation of serum‐free fatty acid levels that contribute to liver pathology (Sakane *et al*, 2021).

In contrast to the studies listed above, Kuramoto *et al* described a different non‐cell‐autonomous mechanism whereby the autophagic machinery regulates systemic energy metabolism through a non‐degradative action. This indicates that besides metabolite‐mediated inter‐organ communication, autophagic machinery exerts its non‐cell‐autonomous function also through a secretion of bioactive molecules such as hormones. The authors demonstrated that hyperactive autophagy protein Becn1 promotes the secretion of adiponectin from white adipose tissue through secretory vesicles. This adipose‐derived metabolic hormone in turn controls energy homeostasis through activation of AMPK and peroxisome proliferator‐activated receptor α signaling pathways. The molecular mechanism of secretion depends on exocyst assembly rather than on autophagosome vesicles that is facilitated by the adipose tissue‐specific upregulation of Becn1 activity. This results in improved systemic insulin sensitivity, glucose tolerance, and lipid metabolism in non‐adipose tissue metabolic organs, implicating the importance of this pathway in obesity and type II diabetes (Kuramoto *et al*, 2021).

In summary, these results demonstrate that the autophagic machinery controls different physiological outcomes in different metabolic tissues (Fig 4). Altogether these studies highlight autophagy as a critical signaling pathway broadcasting signals systemically between different tissues to modulate homeostatic and pathologic state of the organisms.

## Conclusions

In this review, we highlight the role of autophagy in facilitating secretion of various molecules that are mediating intercellular and interorgan crosstalk in health and disease. We show that autophagy is important in mediating homeostasis through removal of dysfunctional material as well as being critical in orchestrating complex signaling and metabolic networks. It offers support to stressed cells (e.g., metabolic switch in cancer cells, reduction of oxidative stress by clearance of damaged mitochondria) and supports tissue biosynthetic and catabolic processes. As such, autophagy can be regarded as having cytokine‐ and vesicle‐secreting and nutrient‐generating roles, shaping cell fate decisions, tissue microenvironment, and supporting systemic metabolic well‐being. Notably, lysosomal exocytosis is an alternative pathway to secretory autophagy and obviously autophagy contributes to lysosomal content (Martins *et al*, 2014; Rovira *et al*, 2022). In the future, it will be even more important to clearly differentiate the contribution of the two pathways to the secretion of autophagosomal cargo versus lysosomal cargo, as demonstrated by Martins *et al*. Currently known mechanistic insights into lysosomal exocytosis would be valuable for a further discussion in a separate review. Interestingly, the roles of degradative and secretory autophagy lead to quite diverse outcomes in terms of inflammation. While cell‐intrinsic autophagy suppresses inflammation through inflammasome degradation or inhibition leading to reduced IL‐1β production (Harris *et al*, 2011; Shi *et al*, 2012; Saitoh & Akira, 2016; Ardianto *et al*, 2022), cell‐extrinsic autophagy promotes inflammation by secretion of IL‐1β after inflammasome activation. Similar opposing functions of cell intrinsic versus extrinsic autophagy have also been observed in cancer. These controversies are difficult to resolve and may have to do with the diverse impacts that autophagy can have on a cell, including cellular metabolism and differentiation leading to different signaling outcomes (Box 2). Likewise, it is challenging to explain these disparities through evolution, since exo/endocytosis and autophagy are highly conserved, and all exist in the last common eukaryotic ancestor (Box 2). Nevertheless, since endo‐ and exocytosis‐like pathways can be found in prokaryotes and Archaea, it is plausible that secretory autophagy has taken on a distinct function, however, due to lack of evidence it currently appears to represent a compensatory pathway, for example when lysosomes are not competent (mimicked by addition of Bafilomycin A1). Pathway redundancy in specific cases mentioned throughout this review remains unclear and requires further investigation, however, it is plausible that stress (e.g., cancer, inflammation, obesity) serves as one of the activators of secretory autophagy, in contrast to the homeostatic function of exo/endocytosis and intracellular autophagy. It is plausible that under physiological conditions, autophagy acts to prevent excessive inflammation by degrading IL‐1β, while under pathological conditions, autophagy acts to release IL‐1β to trigger protective immune responses (Cavalli & Cenci, 2020). If this hypothesis is experimentally proven, it would demonstrate that autophagy critically responds to upstream signals and depending on the strength of the signal it contains or promotes inflammation through distinct pathways. This might be expanded to more cytokines than just IL‐1β. In addition, it might be that the fate between cell‐intrinsic versus cell‐extrinsic is determined by autophagosome‐interacting factors or health of the lysosomal compartment. For example, when lysosomes from aged organisms contain lipofuscin, they are unable to fuse to the autophagosomes and the cargo would be expelled instead. Another scenario may be when too much material is in need of degradation and both systems need to function side‐by‐side. Further research is needed to confirm these hypotheses. In the future, it will be critical to better discern between direct versus indirect outcomes of non‐cell‐autonomous autophagy through understanding the underpinning secretory mechanisms with a combination of genetic, pharmacological, and biochemical approaches (Box 2). It will be important to further study the autophagy‐derived secretome, as these candidates might represent important non‐invasive biomarkers for disease progression, or drug targets. Furthermore, some of the key questions that remain to be answered also revolve around the mechanism of fusion of the secretory autophagosome with the plasma membrane and degradation of the inner autophagosome membrane during the process. Addressing these questions will be instrumental to exploit the function of non‐cell autonomous autophagy in disease management.

Box 2 **In need of answers**

- What are the upstream homeostatic or pathological signals that trigger a switch from cell‐intrinsic to cell‐extrinsic autophagy? How could these help explain the differential roles of the two autophagic pathways?
- Why has secretory autophagy evolved in the first place? Does it have a distinct function to exocytosis?
- What is the mechanism of fusion of the autophagosome with the plasma membrane? Does the inner autophagosome membrane get degraded during fusion and how?
- How can we more clearly differentiate between direct versus indirect contribution of autophagy to the cellular secretome in future studies?

## Author contributions

**Klara Piletic:** Conceptualization; funding acquisition; visualization; writing – original draft; writing – review and editing. **Ghada Alsaleh:** Conceptualization; supervision; visualization; writing – original draft; writing – review and editing. **Anna Katharina Simon:** Conceptualization; supervision; funding acquisition; visualization; writing – original draft; writing – review and editing.

## Disclosure and competing interests statement

The authors declare that they have no conflict of interest.
